# First Comprehensive Report of Clinical *Fusarium* Strains Isolated in the State of Sao Paulo (Brazil) and Identified by MALDI-TOF MS and Molecular Biology

**DOI:** 10.3390/microorganisms8010066

**Published:** 2019-12-31

**Authors:** Mario Henrique Paziani, Ludmilla Tonani Carvalho, Marcia de Souza Carvalho Melhem, Margarete Teresa Gottardo de Almeida, Maria Emilia Nadaletto Bonifácio da Silva, Roberto Martinez, Cledir Santos, Marcia Regina von Zeska Kress

**Affiliations:** 1 Faculdade de Ciencias Farmaceuticas de Ribeirao Preto, Universidade de Sao Paulo, Ribeirao Preto, SP 14040-900, Brazil; mariopaziani@usp.br (M.H.P.); ludmilla@fcfrp.usp.br (L.T.C.); nadaleto@usp.br (M.E.N.B.d.S.); kress@fcfrp.usp.br (M.R.v.Z.K.); 2 Department of Chemical Sciences and Natural Resources, BIOREN-UFRO, Universidad de La Frontera, Temuco 4811-230, Chile; 3 Faculdade de Medicina, Universidade Federal de Mato Grosso do Sul, Campo Grande, MS 79070-900, Brazil; melhemmr@uol.com.br; 4 Secretary of Health, Government of Sao Paulo State, Sao Paulo, SP 01246-000, Brazil; 5 Faculdade de Medicina de Sao Jose do Rio Preto-FAMERP, Sao Jose do Rio Preto, SP 15090-000, Brazil; margarete@famerp.br; 6 Faculdade de Medicina de Ribeirao Preto, Universidade de Sao Paulo, Ribeirao Preto, SP 14040-900, Brazil; rmartine@fmrp.usp.br

**Keywords:** *Fusarium* species complex, *Fusarium* species, fusariosis, molecular identification, MALDI-TOF MS

## Abstract

The aim of this study was to compare the performance of matrix-assisted laser desorption/ionization time-of-flight mass spectrometry (MALDI-TOF MS), phenotypic and molecular methods for the identification of *Fusarium* species complexes isolated from clinical cases in the State of Sao Paulo (Brazil) between the years 2001 and 2017. Sequencing of ITS region of ribosomal DNA and elongation factor 1 alpha gene (ET1α) were used as reference method in the analysis of a total of 108 *Fusarium* spp. clinical strains isolated from human hosts with superficial and systemic infections. Agreement between MALDI-TOF-MS and molecular data was observed for 97 out of 108 clinical isolates (89.8%), whereas five (4.6%) and six (5.5%) clinical isolates were misidentified and were not identified by MALDI-TOF MS, respectively. ITS region sequences and MALDI-TOF MS mass spectra identified and grouped correctly most of *Fusarium* clinical isolates at species complex level. This investigation highlights the potential of MALDI-TOF MS technique as a fast and cost-efficient alternative for clinical *Fusarium* identification. However, MALDI-TOF MS requires a more accurate and larger database. This work is the first comprehensive report for *Fusarium* population, based on phenotypic analyses, proteomic profile by MALDI-TOF and phylogenetic analyses of *Fusarium* species complexes isolated from clinical cases in the State of Sao Paulo, Brazil.

## 1. Introduction

*Fusarium* spp. are filamentous fungi isolated from a wide variety of substrates such as soil, plant and water, mainly in tropical regions. They are mainly primary plant pathogens, although some *Fusarium* species can live within plants tissues as endophytic fungi [1,2,3,4,5,6,7]. The great ability to survive in different climates makes this fungal species highly adaptable to different environments. *Fusarium* spp. asexual reproduction results in the formation of micro- and macro-conidia, which are responsible for the dispersion in the environment and infections in the host [1,2,8,9].

In recent years, *Fusarium* spp. gained importance in human health. There is an increasing incidence of infections related to immunosuppressed patients (e.g., elderly, AIDS, solid organ, and bone marrow transplantation; oncological, hematological, and autoimmune diseases) [10,11]. The number of subcutaneous fusarioses has increased at alarming levels, making fusariosis, after aspergillosis, the second most common filamentous fungal infection in patients with hematologic neoplasms [12,13,14].

Fusariosis may progress from superficial infection to a generalized severe infection in humans, with a mortality rate of 50%, 40%, and 37.5% by disseminated, skin, and pulmonary fusariosis, respectively [8,10,11]. Moreover, 15% to 27% of patients with keratitis by *Fusarium* spp. present ulcers and high corneal damage [8], and have also been reported to cause mycetoma in patients of tropical countries [15]. However, failures in the correct identifications result in few information about the species involved in these infections cases [16,17].

The genus *Fusarium* houses more than 200 species divided into 22 species complexes, which are differentiated by morphology, host association, and mainly by molecular features [16,17]. Species complex (SC) of *Fusarium solani* (FSSC), *F. oxysporum* (FOSC), and *F. fujikuroi* (FFSC) present the greatest clinical importance [18,19].

Classical morphology identification including macro- and micromorphology analysis is a time-consuming method and is not a completely straight forward taxonomical methodology. However, it is still used as an important way to delimitate the boundary of fungal species within a genus, mainly in case of discordant information [20].

Molecular methods are the gold standard of fungal taxonomy which generates accurate identification. It represents a useful strategy once presents fast results, and high sensitivity and specificity in comparison with the conventional methods of fungal identification [21,22].

Matrix-assisted laser desorption/ionization time-of-flight mass spectrometry (MALDI-TOF MS) has been used as a routine technique for fungal identification at species level, and represents an important phenotypic methodology used for fungal identification [23,24,25]. In addition, MALDI-TOF MS has been used to identify both yeasts and filamentous fungal species and other microbial taxonomic groups [26,27].

The databases of the software associated with a MALDI-TOF MS is enriched by reference spectra of the most commonly encountered yeasts in the laboratory, such as *Candida* spp. and *Cryptococcus* spp. However, relatively few data are currently available regarding the identification of filamentous fungi [28].

The aim of this study was to compare for the first time the performance of MALDI-TOF MS, phenotypic analyses and molecular methods on the identification of *Fusarium* species complex clinical strains isolated from biological samples of patients with superficial and systemic fusariosis, between the years 2001 and 2017 from Sao Paulo State, Brazil.

## 2. Material and Methods

### 2.1. Fungal Strains and Culture Conditions

The clinical strains from this study were isolated from biological samples (toenail, fingernail, skin, blood, and peritoneal fluid) of patients with superficial and systemic fusariosis, between the years 2001 and 2017 in the State of Sao Paulo, Brazil. A total of 108 clinical isolates of *Fusarium* spp. were studied. Among them, 39 clinical isolates were obtained from Sao Jose do Rio Preto (20°49′13″ S, 49°22′47″ W), 55 clinical isolates from Ribeirao Preto (10′36″ S, 47°49′15″ W), and 14 clinical isolates from Sao Paulo city (32′56″ S, 46°38′20″ W). The control strains were *Fusarium keratoplasticum* INCQS 40099 (ATCC 36031) and *F. oxysporum* INCQS 40144 (ATCC 48112).

Clinical isolates were cultivated on Potato Dextrose Agar (PDA) medium (Becton, Dickinson Company, Franklin Lakes, NJ, USA) by incubation at 28 °C for 3 to 5 days. The microconidia were collected with sterile phosphate buffered saline (PBS), pH 7.4, and filtered with sterile Miracloth (Merck Millipore, Burlington, MA, USA). Microconidia were counted with a hemocytometer and the microconidia suspensions were prepared with sterile PBS.

The present study was approved by the ethics committee Comite de Etica em Pesquisa (CEP) of the Faculdade de Ciencias Farmaceuticas de Ribeirao Preto, Universidade de Sao Paulo (code n° 0450-CAAE 70079517.8.00005403, approved on 4 September 2017). All clinical isolates were deposited at the Chilean Culture Collection of Type Strains (http://ccct.ufro.cl), which is member of the World Federation of Culture Collection under the registration number WDCM 1111.

### 2.2. Phenotypic Identification of *Fusarium* Genus

*Fusarium* identification at genus level was performed by classical method. The morphological identification of fungi was based on the taxonomic key and guides available for *Fusarium* species [29]. Briefly, the fungal colonies were grown on PDA medium at 28 °C for 3 to 5 days. The colony color (front and reverse side) were observed. Micromorphological analysis was performed by microculture method. Microconidia of clinical isolates were inoculated on a thin layer of PDA culture medium on a sterile microscope slide. A sterile coverslip was placed above the inoculum and incubated at 28 °C for 48 h in humid chamber. The coverslips with the fungal cells were mounted on microscopic slides with lactophenol blue cotton solution and the conidia, conidiophores and hyphae were assessed by optical microscopy (400×, Observer Z 1—Carl Zeiss Jena, Germany). The images were captured with HDCE-X5 camera (Carton Optical Industries Ltd., Tokyo, Japan) and ScopImage 9.0 software (Lawlogix, Phoenix, AZ, USA).

### 2.3. Molecular Identification of *Fusarium* spp.

Molecular identification of *Fusarium* species complex and species were performed by sequencing the internal transcribed spacer (ITS) region of ribosomal DNA [29] and the elongation factor 1 alpha (EF-1α) gene, respectively [30]. Mycelia were produced by inoculating microconidia of clinical isolates into Sabouraud–dextrose broth media and incubated at 28 °C at 120 rpm (MAX Q 4000 Benchtop Orbital Shaker Incubator (Thermo Fisher Scientific, Waltham, MA, USA) for 3 days. Genomic DNA was extracted from the mycelia as previously described elsewhere [31].

The polymerase chain reactions (PCR) amplifications were carried out with TransTaq^®^ DNA Polymerase High Fidelity (TransGen Biotech, Beijing, China) and primers ITS1 (5′ TCC GTA GGT GAA CCT GCG G 3′) and ITS4 (5′ TCC TCC GCT TAT TGA TAT GC 3′) [29]; and EF-1α forward (5′ ATG GGT AAG GA(A/G) GAC AAG AC 3′) and EF-1α reverse (5′ GGA (G/A)GT ACC AGT (G/C)AT CAT GTT-3’) [30]. Purified PCR products were sequenced with the respective primers in ABI 3730 DNA Analyzer (Life Technologies–Applied Biosystems, Waltham, MA, USA).

Sequences were analyzed and compared with GenBank database at the National Center for Biotechnology Information (NCBI). The evolutionary historical phylogenetic analysis was inferred by the unweighted-pair group method with arithmetic averages (UPGMA) with the concatenated sequences ITS and EF-1α that generated a dendrogram based on *p*-distance. Phylogenetic analyzes were performed using the MEGA 6.0 software (www.megasoftware.net/). The percentage of replicate trees in which the associated taxa clustered together in the bootstrap test (1000 replicates) is shown next to the branches. The evolutionary distances were computed using the Maximum Composite Likelihood method.

### 2.4. MALDI-TOF MS Identification of *Fusarium* spp.

MALDI-TOF MS analysis was developed as previously described elsewhere [30] with modifications. Briefly, microconidia of *Fusarium* spp. clinical isolates were inoculated on PDA medium (Acumedia, Lansing, MI, USA) and incubated at 28 °C for 5 days. The fungal cells were transferred to 1.5-mL microtubes containing 500 µL of ethanol 70% (*v*/*v*) (Merk KGaA, Darmstadt, Germany) and glass beads (Sigma-Aldrich, St. Louis, MO, USA).

Microtubes were vortexed for 5 min and sonicated for 10 min twice to break down the fungal cell wall and exposing the proteins. Each sample (1 µL) was transferred to the surface of stainless steel MALDI sample plate (Bruker Daltonics, Bremen, Germany) and air dried. Once dry, MALDI-TOF alpha-cyano-4-hydroxycinnamic acid matrix solution (1 µL) (CHCA, Fluka, Buchs, Switzerland) saturated in a solution with 33% (*v*/*v*) ethanol, 33% (*v*/*v*) acetonitrile, 31% (*v*/*v*) H_2_O and 3% (*v*/*v*) trifluoroacetic acid was gently mixed to each sample on stainless steel MALDI sample plate and air-dried again. Mass spectra were obtained by a MALDI-TOF MS Autoflex Speed (Bruker Daltonics, Bremen, Germany) equipped with a smart beam laser source (355 nm). All samples were deposited in duplicate on the stainless steel MALDI sample plate.

Analyzes were performed in linear and positive modes. Each spectrum was collected as an average of 1200 laser shots with enough energy to produce good spectra without saturation in the range of *m*/*z* from 2000 to 10,000 Da. Before analyzes, the equipment was externally calibrated with the protein calibration standard I (Bruker Daltonics, Bremen, Germany) containing insulin, ubiquitin, cytochrome C, and myoglobin. Fungal identification and statistical clustering were carried out with the software MALDI Biotyper Compass 4.1 (Bruker Daltonics, Bremen, Germany) in the range of *m*/*z* 2000 to 20,000.

The acquired spectra were compared with those spectra archived in the software library for filamentous fungi of MALDI Biotyper Compass 4.1 (Bruker Daltonics, Bremen, Germany). Results of fungal identification were presented as logarithmic scores between 0.000 and 3.000. Scores above 1.700 were considered high similarity. The similarity between each *Fusarium* spp. clinical isolate spectrum is expressed as relative or absolute matching of the mass signal. Dendrogram of spectral similarity were finally obtained by agglomerative clustering algorithm with the software MALDI Biotyper Compass 4.1 (Bruker Daltonics, Bremen, Germany).

## 3. Results

A total of 108 *Fusarium* clinical strains were identified based on classical (macro- and micromorphology), molecular, and MALDI-TOF MS methods. Regarding the macromorpholological features, the colonies colors were yellow, orange, beige, dark purple or lilac. The micromorphological structures were conidiophores arising from septaded hyaline hyphae and abundant macroconidia which presented straight, with parallel walls, and variable septation. Based on these results, all isolates were identified within the *Fusarium* genus.

Based on the results of ITS region sequencing it was possible to identify the 108 clinical isolates into four different *Fusarium* species complexes. FSSC is the most abundant with 97 (89.8%) clinical isolates, followed by FOSC with 9 (8.3%) clinical isolates, and FDSC and FFSC with 1 (0.9%) and 1 (0.9%) clinical isolates, respectively (Table 1 and Table S1). *Fusarium* species were determined by the sequencing of EF-1α gene.

**Figure 1:**
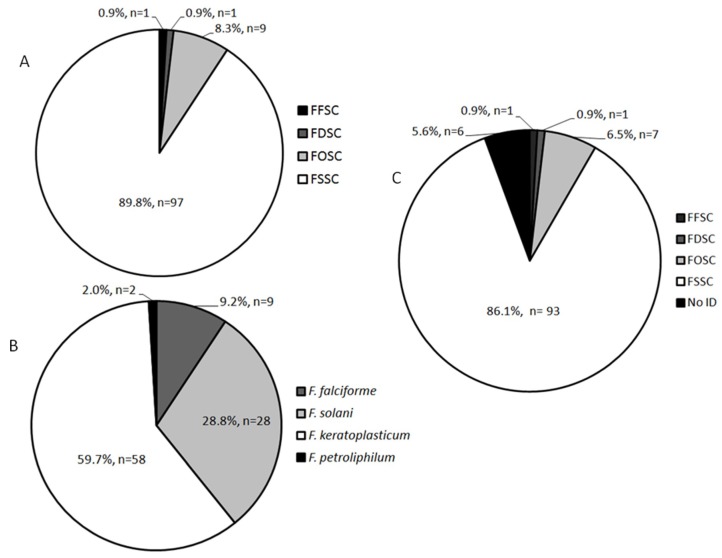
Graphical summary of molecular (ITS and EF-1α) and matrix-assisted laser desorption/ionization time-of-flight mass spectrometry (MALDI-TOF MS) identification of *Fusarium* species complexes clinical isolates (*n* = 108). (**A**) ITS sequencing identification. (**B**) *Fusarium* species of FSSC (sequencing of EF-1α). (**C**) MALDI-TOF MS identification. FSSC: *Fusarium solani* species complex; FOSC: *F. oxysporum* species complex; FFSC: *F. fujikuroi* species complex; FDSC: *F. dimerum* species complex.

**Figure 2:**
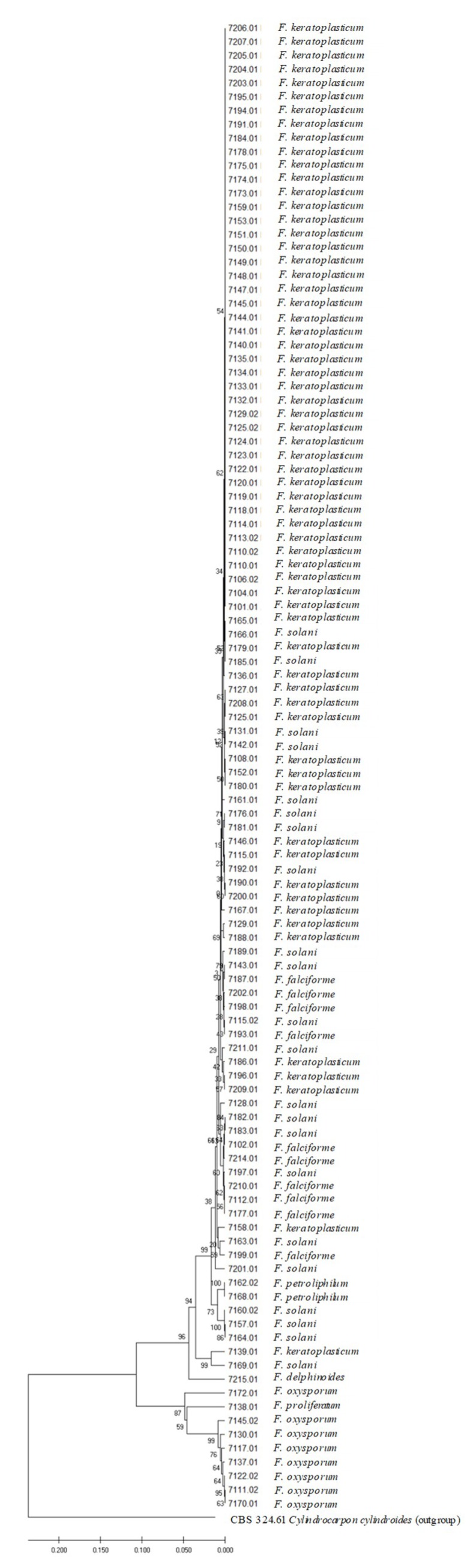
Dendrogram based on concatenated sequences of ITS and EF-1α from 108 *Fusarium* spp. clinical isolates. In the tree nodes are the percentage of the bootstrap. Scale bar indicates nucleotide substitutions per site. FSSC: *Fusarium solani* species complex; FOSC: *F. oxysporum* species complex; FFSC: *F. fujikuroi* species complex; FDSC: *F. dimerum* species complex. Outgroup: *Cylindrocarpon cylindroides* strain CBS 324.61.

Among FSSC clinical isolates are *F. keratoplasticum* (58; 59.7%), *F. solani* (28; 28.8%), *F. falciforme* (9; 9.2%), and *F. petroliphilum* (2; 2.0%) (Figure 1). In the FOSC, all clinical isolates (*n* = 9) are *F. oxysporum*, whereas in FFSC (*n* = 1) and FDSC (*n* = 1) the clinical isolates are *F. proliferatum* and *F. delphinoides*, respectively. In addition, the evolutionary relationship among the clinical isolates is shown in the Figure 2. The UPGMA tree obtained by the concatenation of ITS and EF-1α sequences split the 108 clinical isolates into 2 different clades. All FSSC plus FDSC were grouped in the same clade and all FOSC plus FFSC in another same group (Figure 2).

**Figure 3:**
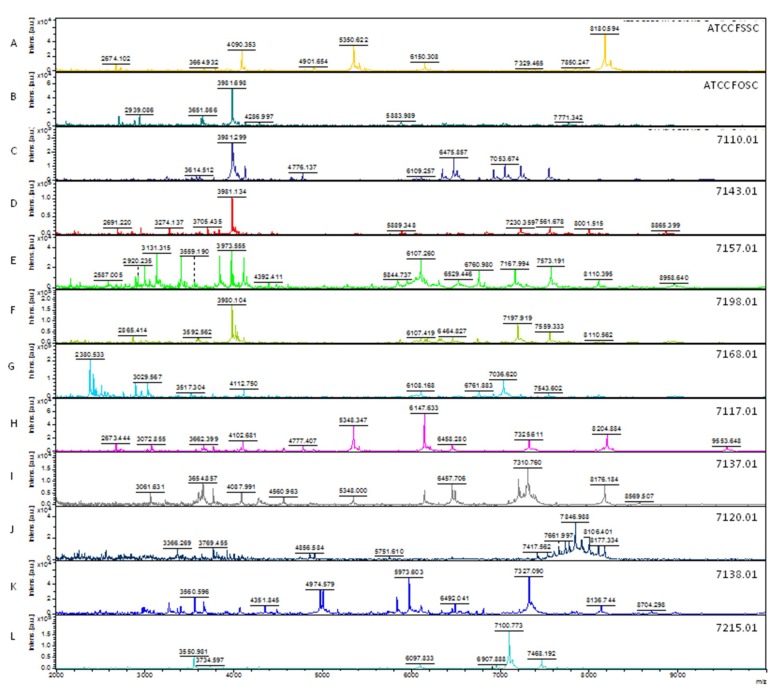
Main MALDI-TOF mass spectra profiles for *Fusarium* species complexes. (**A)** FSSC strain spectrum *(F. keratoplasticum—*ATCC 36031)*;* (**B**) FOSC strain spectrum (*F. oxysporum*—ATCC 48112); (**C**–**G**) FSSC clinical isolates spectra; (**H**–**J**) FOSC clinical isolates spectra; (**K**) FFSC clinical isolate spectrum; (**L**) FDSC clinical isolate spectrum. At the right of the panel is indicated the ID of each strain and/or clinical isolate. Spectra are presented in the range from 2000 to 10,000 Da, where the main mass peaks are observed.

Based on the results of MALDI-TOF MS analysis it was possible to identify 102 out of 108 *Fusarium* spp. clinical isolates. Ninety-three (86.2%) clinical isolates were identified within the FSSC, 7 (6.5%) within the FOSC, 1 (0.9%) within the FFSC, 1 (0.9%) within the FDSC, and 6 (5.5%) were not identified (Table 1 and Table S1). Overall, five different mass spectra profiles were observed for *F. solani* species complex clinical isolates and three different mass spectra profiles for *F. oxysporum* species complex clinical isolates (Figure 3).

**Figure 4:**
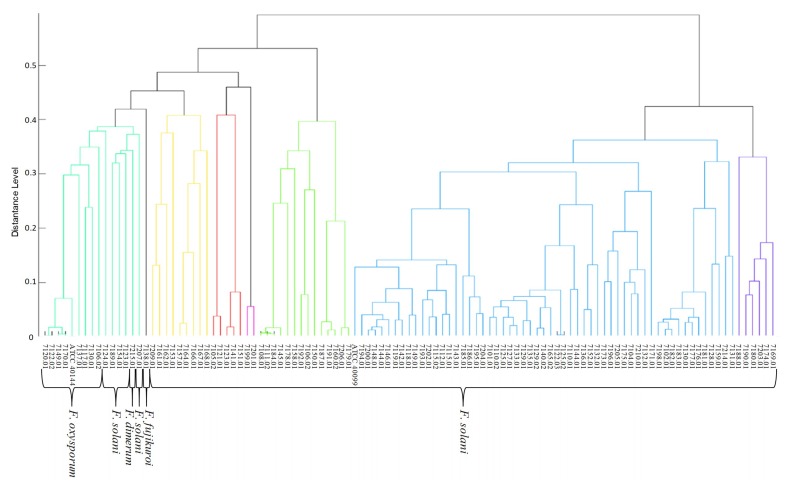
Phenotypic dendrogram based on MALDI-TOF MS spectra, obtained in the range from 2000 to 20,000, for *Fusarium* species complexes. Dendrogram of spectral similarity were obtained by agglomerative clustering algorithm with the software MALDI Biotyper Compass 4.1. C to G—mass spectra profiles of FSSC clinical isolates and control strain.

MALDI-TOF spectral clustering grouped FOSC and FFSC clinical isolates into the same clade (Figure 4). In a different clade are grouped together all FOSC clinical isolates, 4 FSSC clinical isolates (LMC7189.01, LMC7154.01, LMC7123.01, and LMC7207.01) and 1 FDSC clinical isolate (LMC7215.01). The other FSSC clinical isolates were clustered in six different clades (Figure 2).

Analysis by molecular sequencing (ITS region and EF-1α) led to the identification of 98 (100%) clinical isolates as FSSC (Table 1), whereas analysis by MALDI-TOF MS led to the identification of 91 (92.8%) clinical isolates as FSSC. Among 7 clinical isolates identified as FSSC by ITS sequencing, 2 clinical isolates were identified as *F. oxysporum* by MALDI-TOF MS and others 5 clinical isolates were not identified (Table 1 and Table S1).

Among the FOSC clinical isolates, based on the ITS region sequencing it was possible to identify 8 clinical isolates, where 4 (57.1%) matched with MALDI-TOF MS results. However, 3 clinical isolates of FOSC were identified only by MALDI-TOF MS (Table 1). For FFSC and FDSC, both methodologies matched the same clinical isolate identification. Six clinical isolates (2 FOSC and 4 FSSC) were not identified by MALDI-TOF MS (Table 1 and Table S1). Overall, comparing both techniques, molecular sequencing was more reliable than the MALDI-TOF technique.

## 4. Discussion

*Fusarium* is a large and diverse fungal genus with great importance in agriculture, human health, and economy. The species are high mycotoxin producers and pathogens to plants and humans [32,33]. *Fusarium* spp. is the second filamentous fungi causative agent of infection in humans after *Aspergillus* spp. The mortality prognosis of disseminated fusariosis is ~80% [17].

MALDI-TOF MS is a rapid spectral technique and is listed as an alternative method to identify fungi. It is based on fingerprinting of large organic molecules (e.g., ribosomal proteins) that identifies the fungal species at specific spectral level [20,23,25]. Until now, few studies have been published with regard to the identification of a collection of clinical isolates of *Fusarium* species complex by both MALDI-TOF MS and molecular methods [34,35,36]. The present study is the first comprehensive report for *Fusarium* species complex clinical isolates based on phenotypic features, phylogenetic analyses, and proteomic profile by MALDI-TOF MS of clinical strains isolated across the State of Sao Paulo, Brazil.

The performance of MALDI-TOF MS identification compared with classical and molecular methods was evaluated. A total of 108 clinical isolates were included in this investigation. According to the classical method (macro and micromorphology) it was possible to identify all clinical isolates into the genus *Fusarium*. The classical method (macro and micromorphology) is commonly employed as the preliminary methodology for *Fusarium* spp. identification. However, it is usually time-consuming and restricted by the unpredictable and subjective character of phenotypic features, which are immediately affected by culture conditions [21].

Besides classical methods, polymerase chain reaction (PCR) and nucleotide sequence analysis is an identification tool for fungi. Molecular techniques are the most frequently identification methods because of species specificity of this region. The technique is known to provide good resolution at the sub-species level, and thus sequence analysis is a superior choice for phylogeny in *Fusarium* species complexes [13,36,37].

Due to the specificity of *Fusarium* species complex, the sequencing of ITS region of rDNA is most frequently studied to identify *Fusarium* spp. clinical isolates. In addition, ITS sequencing is known to provide good resolution and a superior choice for phylogenetic studies in the species complex [13,37,38,39]. However, the high-throughput sequencing of *Fusarium* spp. communities has been limited by the lack of primers for genus-specific targeting regions with high discriminatory power at the species level [40]. As a matter of consequence, the identification based on the sequencing of ITS region and others regions, such as EF-1α, has provided an alternative approach for higher detection and identification of *Fusarium* spp. [41,42,43].

In the present study, sequencing of ITS region identified *Fusarium* spp. clinical isolates into 4 different *Fusarium* species complex. Most of the clinical isolates were identified within the FSSC (97; 89.8%) and the remaining clinical isolates were divided within the FOSC (9; 8.3%), FFSC (1; 0.9%), and FDSC (1; 0.9%). The EF-1α is another gene which facilitates the identification of fungal species based on DNA sequencing. Here, EF-1α gene identified the 7 species *F. keratoplasticum*, *F. solani*, *F. falciforme*, *F. petroliphilum*, *F. oxysporum*, *F. proliferatum*, and *F. delphinoides*.

To improve the correct identification, the sequences of *Fusarium* spp. genes are available in databases such as *Fusarium*-ID (http://isolate.fusariumdb.org/blast.php) and *Fusarium* MLST Database (www.westerdijkinstitute.nl/fusarium) [40,44].

Molecular biology is recognized as the gold standard method for fungal identification [44,45], but it takes about 4 days in the clinical routine laboratory setting. Moreover, it is also considered expensive and needs skilled microbiologists [45,46]. On the other hand, MALDI-TOF MS is a fast spectral technique and is listed as a suitable method to identify fungi [20,25,47]. Thus, in this study, the identification of the clinical isolates by this faster method was proceeded.

According to MALDI-TOF MS results, 94.4% (*n* = 102) of the clinical isolates were identified at species complex level. Among the identified clinical isolates, MALDI-TOF MS was able to identified 2 clinical isolates at species level, *F. proliferatum* (FFSC) and *F. dimerum* (FDSC). However, results obtained from MALDI-TOF MS showed divergences when compared with results obtained from ITS sequencing method.

Among 102 clinical isolates identified by MALDI-TOF MS, three were identified within the FOSC, while by ITS sequence these isolates were identified within the FSSC; and two isolates were identified by MALDI-TOF within the FSSC, whereas by ITS sequence these isolates were identified within the FOSC.

In conclusion, in the present study, 97 (89.9%) out of 108 clinical isolates were correctly identified by MALDI-TOF MS; whereas 5 clinical isolates (4.6%) were misidentified by this spectral technique and 6 clinical isolates (5.5%) were not identified. In addition, MALDI-TOF MS was not able to discriminate the species of FSSC.

MALDI-TOF MS divergences in fungal identification and its inability to discriminate the species of FSSC are attributed to the limited database currently provided by the MALDI-TOF MS manufacturers [48,49]. However, these divergences in strain matching are compensated by the low cost and speed in obtaining results [45,46,48]. *Fusarium* species complexes are diverse and different environmental condition can alter the metabolic pathways and rearranging the biosynthetic mechanisms in order to protect themselves against cell damage and death. Due to this, it presents high plasticity of gene regulation and expression which alter the pattern of the spectral mass [41,47,50,51,52], which makes this analysis more complex and requires more detailed spectra stored in databases.

Identification of *Fusarium* genus by MALDI-TOF MS is not yet accurately investigated due to low number of representative taxa in databases [32,53]. Our study identified 12 different spectral mass patterns, from which six within the FSSC and four within the FOSC. This result shows how diverse is this fungal genus and the importance in registering in the database different spectral mass patterns of the same species isolated from different geographical regions and submitted to different growth conditions [32].

In the present study, the MALDI-TOF technique was not able to discriminate the whole evaluated fungal species of the FSSC. In contrast, a previous study Oliveira and collaborators [32] successfully identified species of the clinical fungal genus *Sporothrix* by MALDI-TOF MS. The authors have established an accurate in-house MALDI-TOF spectral database, which is comprehensive in terms of *Sporothrix* MALDI-TOF spectra number and geographical distribution of strain origins. Moreover, all reference spectra available in the mentioned in-house database were previously validated by molecular biology analysis.

This effort in the build-up of an in-house database is substantial and worthwhile. Matos and collaborators [54] have further used the same above mentioned in-house database for the identification by MALDI-TOF MS of a *Sporothrix brasiliensis* strain isolated from a subconjunctival infiltrative lesion in immunocompetent patient. According to the authors, MALDI-TOF MS technique was as good as the partial sequence of genes (calmodulin, beta-tubulin and chitin synthase, for example) for the identification of *S. brasiliensis*.

Based on the information obtained in the present study, and evaluating the information available for identification of other fungal taxonomic group by MALDI-TOF MS [32,54], it is possible to understand that MALDI-TOF limitation in identifying species of the FSSC is mainly because this analytical technique requires a more accurate and larger database for fungal identification.

Establishing an in-house database is a good way to overcome the limitation associated with using MALDI-TOF MS in fungal identification. For future studies on clinical *Fusarium* identification by MALDI-TOF MS, the data generated here can be used in the establishment of an accurate in-house MALDI-TOF spectral database.

## 5. Conclusions

This work is the first comprehensive report for populations based on the phenotypic analyses, proteomic profile by MALDI-TOF, and phylogenetic analyses of *Fusarium* species complex isolated from clinical cases in the State of Sao Paulo, Brazil. FSSC was the most frequent *Fusarium* clinical complex in Sao Paulo State, followed by FOSC, FDSC, and FFSC. The results of ITS region sequencing and MALDI-TOF MS mass spectra led to the identification and grouping of most *Fusarium* clinical strains at species complex. MALDI-TOF MS identification has shown 89.8% concordance with molecular methods, highlighting its potential on filamentous fungi identification as a fast and cost-efficient alternative; however, MALDI-TOF MS requires a more accurate and larger database.

## Figures and Tables

**Table 1:** Molecular method (ITS and EF-1α sequencing) and MALDI-TOF MS identification of the 108 *Fusarium* spp. clinical isolates

Method	FSSC	%	FOSC	%	FFSC	%	FDSC	%	No ID	%	Total	%
Molecular	97	89.8	9	8.3	1	0.9	1	0.9	0	0	108	100.0
MALDI TOF MS	93	86.1	7	6.5	1	0.9	1	0.9	6	5.6	108	100.0

FSSC: *Fusarium solani* species complex; FOSC: *F. oxysporum* species complex; FFSC: *F. fujikuroi* species complex; FDSC: *F. dimerum* species complex; No ID: no identification.

## Supplementary Materials

The following are available online at https://www.mdpi.com/2076-2607/8/1/66/s1.
