# Supplementary material for: First Comprehensive Report of Clinical Fusarium Strains Isolated in the State of Sao Paulo (Brazil) and Identified by MALDI-TOF MS and Molecular Biology
Source: Microorganisms. 2019 Dec 31;8(1):66. doi: 10.3390/microorganisms8010066 (PMC7022604; doi:10.3390/microorganisms8010066)
Supplement: Supplementary file 1 [file microorganisms-08-00066-s001.pdf]

Table S1: *Fusarium* species identification by ITS and ET1α sequencing, and MALDI-TOF MS. ID, identification; ITS, internal transcribed spacer; EF1α, elongation factor 1 alpha; FSSC, *Fusarium solani* species complex; FOSC, *F. oxysporum* species complex; FDSC, *F. dimerum* species complex; FFSC, *F. fujikuroi* species complex; N/I, non-significant identification; CCCT, Chilean Culture Collection of Type.

Strain code	Surce	MOLECULAR IDENTIFICATION								MALDI-TOF MS		GenBank Accession number		CCCT code
		ITS				EF1α								
		ID	%ID	*e*-value	GenBank Reference	ID	%ID	*e*-value	GenBank reference	ID	*Score*	ITS	EF1α	
**7101.01**	skin	FSSC	100	0.0	MN540869.1	*F. keratoplasticum*	100	0.0	KU933434.1	FSSC	1.410	MN729363	MN854098	19.01
**7102.01**	nail	FSSC	100	0.0	MN555417.1	*F. falciforme*	99	0.0	AB817182.1	FSSC	1.530	MN729364	MN854099	19.02
**7104.01**	nail	FSSC	100	0.0	MN540869.1	*F. keratoplasticum*	100	0.0	KU933434.1	FSSC	1.930	MN729365	MN854100	19.03
**7106.02**	skin	FSSC	100	0.0	MN540869.1	*F. keratoplasticum*	100	0.0	KU933434.1	FSSC	1.450	MN729366	MN854101	19.04
**7108.01**	nail	FSSC	100	0.0	MN540869.1	*F. keratoplasticum*	100	0.0	KU933434.1	FSSC	2.040	MN729367	MN854102	19.05
**7110.01**	nail	FSSC	100	0.0	MN540869.1	*F. keratoplasticum*	100	0.0	KU933434.1	FSSC	1.550	MN729368	MN854103	19.06
**7110.02**	nail	FSSC	100	0.0	MN540869.1	*F. keratoplasticum*	100	0.0	KU933434.1	FSSC	1.780	MN729369	MN854104	19.07
**7111.02**	nail	FSSC	98	0.0	KR527137.2	*F. solani*	99	0.0	DQ247559.1	FSSC	1.990	MN729370	MN854105	19.08
**7112.01**	nail	FSSC	99	0.0	KR527137.1	*F. falciforme*	99	0.0	AB817182.1	FSSC	1.610	MN729371	MN854106	19.09
**7113.02**	skin	FSSC	100	0.0	KR527137.1	*F. solani*	99	0.0	DQ247559.1	FOSC	1.480	MN729372	MN854107	19.10
**7114.01**	nail	FSSC	100	0.0	MN540869.1	*F. keratoplasticum*	100	0.0	KU933434.1	FSSC	1.580	MN729373	MN854108	19.11
**7115.01**	nail	FSSC	98	0.0	KR527137.1	*F. solani*	100	0.0	DQ247559.1	FSSC	1.970	MN729374	MN854109	19.12
**7115.02**	nail	FSSC	100	0.0	MN540869.1	*F. keratoplasticum*	100	0.0	KU933434.1	FSSC	1.580	MN729375	MN854110	19.13
**7118.01**	nail	FSSC	100	0.0	MN540869.1	*F. keratoplasticum*	100	0.0	KU933434.1	FSSC	1.560	MN729376	MN854111	19.14
**7119.01**	skin	FSSC	100	0.0	MN540869.1	*F. keratoplasticum*	100	0.0	KU933434.1	FSSC	1.320	MN729377	MN854112	19.15
**7120.01**	nail	FSSC	100	0.0	MN540869.1	*F. keratoplasticum*	100	0.0	KU933434.1	FSSC	1.430	MN729378	MN854113	19.16
**7122.03**	nail	FSSC	100	0.0	MN540869.1	*F. keratoplasticum*	100	0.0	KU933434.1	FSSC	1.680	MN729379	MN854114	19.17
**7123.01**	skin	FSSC	100	0.0	MN540869.1	*F. keratoplasticum*	100	0.0	KU933434.1	FSSC	1.850	MN729380	MN854115	19.18
**7125.01**	nail	FSSC	100	0.0	MN540869.1	*F. keratoplasticum*	100	0.0	KU933434.1	FSSC	1.660	MN729381	MN854116	19.19
**7125.02**	nail	FSSC	100	0.0	MN540869.1	*F. keratoplasticum*	100	0.0	KU933434.1	FSSC	1.610	MN729382	MN854117	19.20
**7127.01**	nail	FSSC	100	0.0	MN540869.1	*F. keratoplasticum*	99	0.0	KU933434.1	FSSC	1.570	MN729383	MN854118	19.21
**7128.01**	nail	FSSC	99	0.0	KR527137.1	*F. solani*	100	0.0	DQ247559.1	FSSC	1.750	MN729384	MN854119	19.22
**7129.01**	skin	FSSC	100	0.0	MN540869.1	*F. keratoplasticum*	100	0.0	KU933434.1	FSSC	1.430	MN729385	MN854120	19.23
**7129.02**	nail	FSSC	100	0.0	MN540869.1	*F. keratoplasticum*	99	0.0	KU933434.1	FSSC	1.850	MN729386	MN854121	19.24
**7131.01**	nail	FSSC	100	0.0	KR527137.1	*F. solani*	98	0.0	DQ247559.1	FSSC	1.800	MN729387	MN854122	19.25
**7132.01**	nail	FSSC	100	0.0	MN540869.1	*F. keratoplasticum*	100	0.0	KU933434.1	FSSC	1.210	MN729388	MN854123	19.26
**7133.01**	nail	FSSC	100	0.0	MN540869.1	*F. keratoplasticum*	100	0.0	KU933434.1	N/I	-	MN729389	MN854124	19.27
**7134.01**	nail	FSSC	100	0.0	MN540869.1	*F. keratoplasticum*	99	0.0	KU933434.1	FSSC	1.440	MN729390	MN854125	19.28
**7135.01**	nail	FSSC	100	0.0	MN540869.1	*F. keratoplasticum*	100	0.0	KU933434.1	FSSC	1.460	MN729391	MN854126	19.29
**7136.01**	nail	FSSC	100	0.0	MN540869.1	*F. keratoplasticum*	99	0.0	KU933434.1	FSSC	1.850	MN729392	MN854127	19.30
**7139.01**	nail	FSSC	98	0.0	KR527137.1	*F. solani*	99	0.0	DQ247559.1	FOSC	1.460	MN729393	MN854128	19.31
**7140.01**	nail	FSSC	100	0.0	MN540869.1	*F. keratoplasticum*	100	0.0	KU933434.1	FSSC	1.510	MN729394	MN854129	19.32
**7141.01**	nail	FSSC	100	0.0	MN540869.1	*F. keratoplasticum*	100	0.0	KU933434.1	FSSC	2.080	MN729395	MN854130	19.33
**7142.01**	nail	FSSC	100	0.0	KR527137.1	*F. solani*	99	0.0	DQ247559.1	FSSC	1.430	MN729396	MN854131	19.34
**7143.01**	nail	FSSC	100	0.0	KR527137.1	*F. solani*	99	0.0	DQ247559.1	FSSC	1.560	MN729397	MN854132	19.35
**7144.01**	Peritoneal fluid	FSSC	100	0.0	KR527137.1	*F. solani*	99	0.0	DQ247559.1	N/I	-	MN729398	MN854133	19.36
**7145.01**	nail	FSSC	100	0.0	MN540869.1	*F. keratoplasticum*	100	0.0	KU933434.1	FSSC	2.070	MN729399	MN854134	19.37
**7146.01**	nail	FSSC	99	0.0	MN540869.1	*F. keratoplasticum*	100	0.0	KU933434.1	FSSC	1.940	MN729400	MN854135	19.38
**7147.01**	nail	FSSC	99	0.0	MN540869.1	*F. keratoplasticum*	100	0.0	KU933434.1	FSSC	1.730	MN729401	MN854136	19.39
**7148.01**	Peritoneal fluid	FSSC	100	0.0	KR527137.1	*F. solani*	100	0.0	DQ247559.1	FOSC	1.220	MN729402	MN854137	19.40
**7149.01**	nail	FSSC	100	0.0	MN540869.1	*F. keratoplasticum*	99	0.0	KU933434.1	FSSC	1.540	MN729403	MN854138	19.41
**7150.01**	nail	FSSC	99	0.0	MN540869.1	*F. keratoplasticum*	99	0.0	KU933434.1	FSSC	1.660	MN729404	MN854139	19.42
**7151.01**	nail	FSSC	100	0.0	MN540869.1	*F. keratoplasticum*	99	0.0	KU933434.1	FSSC	1.650	MN729405	MN854140	19.43
**7152.01**	nail	FSSC	99	0.0	MN540869.1	*F. keratoplasticum*	100	0.0	KU933434.1	FSSC	1.860	MN729406	MN854141	19.44
**7153.01**	nail	FSSC	100	0.0	MN540869.1	*F. keratoplasticum*	100	0.0	KU933434.1	FSSC	1.460	MN729407	MN854142	19.45
**7157.01**	blood	FSSC	100	0.0	KR527137.1	*F. solani*	99	0.0	DQ247559.1	FSSC	1.860	MN729408	MN854143	19.46
**7158.01**	blood	FSSC	100	0.0	MN540869.1	*F. keratoplasticum*	99	0.0	KU933434.1	FSSC	1.970	MN729409	MN854144	19.47
**7159.01**	blood	FSSC	100	0.0	MN540869.1	*F. keratoplasticum*	100	0.0	KU933434.1	FSSC	2.150	MN729410	MN854145	19.48
**7160.02**	blood	FSSC	100	0.0	KR527137.1	*F. solani*	100	0.0	DQ247559.1	FSSC	1.770	MN729411	MN854146	19.49
**7161.01**	blood	FSSC	100	0.0	KR527137.1	*F. solani*	99	0.0	DQ247559.1	FSSC	1.680	MN729412	MN854147	19.50
**7162.02**	blood	FSSC	99	0.0	KP132225.1	*F. petroliphilum*	100	0.0	MF467468.1	FSSC	1.870	MN729413	MN854148	19.51
**7163.01**	blood	FSSC	100	0.0	KR527137.1	*F. solani*	100	0.0	DQ247559.1	FSSC	1.910	MN729414	MN854149	19.52
**7164.01**	blood	FSSC	100	0.0	KR527137.1	*F. solani*	100	0.0	DQ247559.1	FSSC	1.980	MN729415	MN854150	19.53
**7165.01**	blood	FSSC	100	0.0	MN540869.1	*F. keratoplasticum*	100	0.0	KU933434.1	FSSC	1.660	MN729416	MN854151	19.54
**7166.01**	blood	FSSC	99	0.0	KR527137.1	*F. solani*	100	0.0	DQ247559.1	FSSC	1.320	MN729417	MN854152	19.55
**7167.01**	blood	FSSC	99	0.0	MN540869.1	*F. keratoplasticum*	100	0.0	KU933434.1	FSSC	2.010	MN729418	MN854153	19.56
**7168.01**	blood	FSSC	99	0.0	KP132225.1	*F. petroliphilum*	100	0.0	MF467468.1	FSSC	1.950	MN729419	MN854154	19.57
**7169.01**	blood	FSSC	98	0.0	KR527137.1	*F. solani*	100	0.0	DQ247559.1	FSSC	2.230	MN729420	MN854155	19.58
**7173.01**	nail	FSSC	100	0.0	MN540869.1	*F. keratoplasticum*	100	0.0	KU933434.1	FSSC	1.640	MN729421	MN854156	19.59
**7174.01**	nail	FSSC	100	0.0	MN540869.1	*F. keratoplasticum*	100	0.0	KU933434.1	FSSC	1.980	MN729422	MN854157	19.60
**7175.01**	nail	FSSC	100	0.0	MN540869.1	*F. keratoplasticum*	100	0.0	KU933434.1	FSSC	1.600	MN729423	MN854158	19.61
**7176.01**	nail	FSSC	100	0.0	KR527137.1	*F. solani*	100	0.0	DQ247559.1	FSSC	1.620	MN729424	MN854159	19.62
**7177.01**	nail	FSSC	100	0.0	MN555417.1	*F. falciforme*	100	0.0	AB817182.1	FSSC	1.620	MN729425	MN854160	19.63
**7178.01**	nail	FSSC	99	0.0	MN540869.1	*F. keratoplasticum*	99	0.0	KU933434.1	FSSC	1.570	MN729426	MN854161	19.64
**7179.01**	nail	FSSC	99	0.0	MN540869.1	*F. keratoplasticum*	99	0.0	KU933434.1	FSSC	1.640	MN729427	MN854162	19.65
**7180.01**	nail	FSSC	100	0.0	MN540869.1	*F. keratoplasticum*	100	0.0	KU933434.1	FSSC	2.060	MN729428	MN854163	19.66
**7181.01**	skin	FSSC	100	0.0	KR527137.1	*F. solani*	100	0.0	DQ247559.1	FSSC	1.550	MN729429	MN854164	19.67
**7182.01**	nail	FSSC	100	0.0	KR527137.1	*F. solani*	100	0.0	DQ247559.1	FSSC	1.540	MN729430	MN854165	19.68
**7183.01**	nail	FSSC	100	0.0	KR527137.1	*F. solani*	100	0.0	DQ247559.1	FSSC	1.710	MN729431	MN854166	19.69
**7184.01**	nail	FSSC	99	0.0	MN540869.1	*F. keratoplasticum*	100	0.0	KU933434.1	FSSC	1.750	MN729432	MN854167	19.70
**7185.01**	nail	FSSC	100	0.0	KR527137.1	*F. solani*	100	0.0	DQ247559.1	FSSC	1.570	MN729433	MN854168	19.71
**7186.01**	nail	FSSC	100	0.0	MN540869.1	*F. keratoplasticum*	100	0.0	KU933434.1	FSSC	1.500	MN729434	MN854169	19.72
**7187.01**	nail	FSSC	99	0.0	MN555417.1	*F. falciforme*	100	0.0	AB817182.1	FSSC	1.990	MN729435	MN854170	19.73

**7188.01**	nail	FSSC	99	0.0	MN540869.1	*F. keratoplasticum*	100	0.0	KU933434.1	FSSC	1.550	MN729436	MN854171	19.74
**7189.01**	nail	FSSC	100	0.0	KR527137.1	*F. solani*	100	0.0	DQ247559.1	FSSC	2.130	MN729437	MN854172	19.75
**7190.01**	nail	FSSC	100	0.0	MN540869.1	*F. keratoplasticum*	100	0.0	KU933434.1	FSSC	1.770	MN729438	MN854173	19.76
**7191.01**	nail	FSSC	100	0.0	MN540869.1	*F. keratoplasticum*	100	0.0	KU933434.1	FSSC	1.650	MN729439	MN854174	19.77
**7192.01**	nail	FSSC	100	0.0	KR527137.1	*F. solani*	100	0.0	DQ247559.1	FSSC	2.000	MN729440	MN854175	19.78
**7193.01**	nail	FSSC	99	0.0	MN555417.1	*F. falciforme*	100	0.0	AB817182.1	FSSC	1.730	MN729441	MN854176	19.79
**7194.01**	skin	FSSC	100	0.0	MN540869.1	*F. keratoplasticum*	100	0.0	KU933434.1	FSSC	1.700	MN729442	MN854177	19.80
**7195.01**	nail	FSSC	100	0.0	MN540869.1	*F. keratoplasticum*	100	0.0	KU933434.1	FSSC	1.470	MN729443	MN854178	19.81
**7196.01**	skin	FSSC	100	0.0	MN540869.1	*F. keratoplasticum*	100	0.0	KU933434.1	FSSC	1.520	MN729444	MN854179	19.82
**7197.01**	nail	FSSC	100	0.0	KR527137.1	*F. solani*	99	0.0	DQ247559.1	FSSC	1.410	MN729445	MN854180	19.83
**7198.01**	nail	FSSC	99	0.0	MN555417.1	*F. falciforme*	100	0.0	AB817182.1	FSSC	1.450	MN729446	MN854181	19.84
**7199.01**	nail	FSSC	99	0.0	MN555417.1	*F. falciforme*	99	0.0	AB817182.1	FSSC	1.400	MN729447	MN854182	19.85
**7200.01**	nail	FSSC	100	0.0	MN540869.1	*F. keratoplasticum*	100	0.0	KU933434.1	FSSC	0.990	MN729448	MN854183	19.86
**7201.01**	nail	FSSC	100	0.0	KR527137.1	*F. solani*	100	0.0	DQ247559.1	FSSC	1.880	MN729449	MN854184	19.87
**7202.01**	nail	FSSC	99	0.0	MN555417.1	*F. falciforme*	100	0.0	AB817182.1	FSSC	1.760	MN729450	MN854185	19.88
**7203.01**	nail	FSSC	99	0.0	MN540869.1	*F. keratoplasticum*	100	0.0	KU933434.1	FSSC	1.550	MN729451	MN854186	19.89
**7204.01**	skin	FSSC	100	0.0	MN540869.1	*F. keratoplasticum*	100	0.0	KU933434.1	FSSC	1.270	MN729452	MN854187	19.90
**7205.01**	nail	FSSC	100	0.0	MN540869.1	*F. keratoplasticum*	100	0.0	KU933434.1	FSSC	1.700	MN729453	MN854188	19.91
**7206.01**	nail	FSSC	100	0.0	MN540869.1	*F. keratoplasticum*	100	0.0	KU933434.1	FSSC	2.100	MN729454	MN854189	19.92
**7207.01**	skin	FSSC	100	0.0	MN540869.1	*F. keratoplasticum*	100	0.0	KU933434.1	N/I	-	MN729455	MN854190	19.93
**7208.01**	nail	FSSC	100	0.0	MN540869.1	*F. keratoplasticum*	100	0.0	KU933434.1	FSSC	1.600	MN729456	MN854191	19.94
**7209.01**	nail	FSSC	99	0.0	MN540869.1	*F. keratoplasticum*	100	0.0	KU933434.1	FSSC	1.960	MN729457	MN854192	19.95
**7210.01**	skin	FSSC	100	0.0	MN555417.1	*F. falciforme*	99	0.0	AB817182.1	N/I	-	MN729458	MN854193	19.96
**7211.01**	nail	FSSC	100	0.0	KR527137.1	*F. solani*	99	0.0	DQ247559.1	FSSC	1.530	MN729459	MN854194	19.97
**7214.01**	nail	FSSC	100	0.0	KR527137.1	*F. solani*	99	0.0	DQ247559.1	FSSC	1.680	MN7146.24	MN854195	19.98
**7117.01**	skin	FOSC	100	0.0	JN624906.3	*F. oxysporum*	100	0.0	KM065857.1	FSSC	1.660	MN7146.24	MN854196	19.99
**7122.02**	nail	FOSC	100	0.0	JN624906.3	*F. oxysporum*	100	0.0	KM065857.1	FOSC	1.190	MN7146.25	MN854197	19.100
**7124.01**	nail	FOSC	100	0.0	JN624906.3	*F. oxysporum*	99	0.0	KM065857.1	N/I	-	MN7146.26	MN854198	19.101
**7130.01**	nail	FOSC	99	0.0	JN624906.3	*F. oxysporum*	100	0.0	KM065857.1	FOSC	1.820	MN7146.27	MN854199	19.102
**7137.01**	nail	FOSC	99	0.0	JN624906.3	*F. oxysporum*	100	0.0	KM065857.1	FOSC	1.670	MN7146.28	MN854200	19.103
**7145.02**	skin	FOSC	100	0.0	JN624906.3	*F. oxysporum*	100	0,0	KM065857.1	FSSC	1.170	MN7146.29	MN854201	19.104
**7170.01**	blood	FOSC	100	0.0	JN624906.3	*F. oxysporum*	100	0,0	KM065857.1	FOSC	2.000	MN7146.30	MN854202	19.105
**7172.01**	nail	FOSC	100	0.0	JN624906.3	*F. oxysporum*	100	0,0	KM065857.1	N/I	-	MN7146.31	MN854203	19.106
**7138.01**	nail	FFSC	100	0.0	MK105817.1	*F. proliferatum*	100	0,0	KT239489.1	*F. proliferatum*	1.960	MN729462	MN854204	19.107
**7215.01**	skin	FDSC	100	0.0	NR130680.1	*F. delphinoides*	100	0,0	KX377697.1	*F. dimerum*	1.460	MN708342	MN854205	19.108
